# Giant cell tumor of proximal femur managed by extended curettage with fibular strut allograft using long intramedullary interlocking nail: A case report and literature review

**DOI:** 10.1097/MD.0000000000040960

**Published:** 2024-12-13

**Authors:** Jiashi Song, Bing Liu, Kaipeng Jin, Quan Yao

**Affiliations:** a Department of Orthopedics, Zhejiang Rongjun Hospital, Nanhu District, Jiaxing, Zhejiang, PR China; b Department of Orthopedics, Second Affiliated Hospital, School of Medicine, Zhejiang University, Hangzhou, Zhejiang, PR China; c Orthopedics Research Institute of Zhejiang University, Hangzhou City, Zhejiang Province, PR China.

**Keywords:** extended curettage, fibular strut allograft, Giant cell tumor of the proximal femur, hip joint preserving surgery, intramedullary nail

## Abstract

**Rationale:**

We first report a unique case of proximal femoral Giant cell tumor of bone, a subtrochanteric lesion associated with femoral neck and intertrochanteric involvement. We chose a completely new surgical approach to treat the primary tumor and preserve the hip joint. No cases of this type have ever been reported.

**Patient concerns:**

The patient, a 26-year-old man, came to our hospital for treatment of right hip pain more than 4 months ago, had no family history of similar diseases.

**Diagnoses:**

Based on the imaging results and pathology, a diagnosis of Giant cell tumor of bone was confirmed.

**Interventions:**

Based on the imaging grade and patients’ wishes, the tumor managed by extended curettage and reconstructed with a fibular strut allograft and long intramedullary interlocking nail was used for prophylactic fixation of fractures. The patient did not undergo disuzumab.

**Outcomes:**

After 40 months of follow-up, although the bone defect finally reached bone healing, the hip function was good, and the tumor did not recur, there were signs of internal fixation loosening at 12 months of the surgery.

**Lessons:**

For young patients with imaging grade <3 who need limb salvage, fibular strut allograft and intramedullary nail-fixation are also an alternative treatment option for hip reconstruction after tumor surgery when the lesion involves the entire proximal femur.

**Keypoints**

- We first report a unique case of proximal femoral GCT, a subtrochanteric lesion associated with femoral neck and intertrochanteric involvement. No cases of this type have ever been reported.
- We have chosen a completely new surgical approach for the treatment of primary tumors. Based on the imaging grade and patients’ wishes, the tumor managed by extended curettage and reconstructed with a fibular strut allograft and long intramedullary interlocking nail was used for prophylactic fixation of fractures. After 40 months of follow-up, although the bone defect finally reached bone healing, the hip function was good, and the tumor did not recur, there were signs of internal fixation loosening at 12 months of the surgery.
- Bone defects are often associated with extended curettage of the proximal femur GCT. For young patients with imaging grade less than 3 who need limb salvage, fibular strut allograft and intramedullary nail fixation are also an alternative treatment option for hip reconstruction after tumor surgery when the lesion involves the entire proximal femur. Due to the limited supporting strength of bone grafts and the long healing time of bone, it may be associated with a higher risk of loosening of internal fixation and fracture.

## 1. Introduction

Giant cell tumor of bone (GCT) is a locally aggressive bone tumor, which can locally invade surrounding tissues and metastasized to the lung at a distant distance. Most of them are isolated lesions, the proportion of multi-center lesions is <1%, the age of onset is 20 to 40 years old, which is more commonly seen among female than male, and they are most likely to occur in the epiphysis of the femur, tibia, femur and radius, accounting for about 5% of all primary bone tumors.^[1]^ The main imaging feature of GCT is osteolysis. Currently, a number of scholars agree that the reason of osteolysis caused by GCT is that osteoblast-like mononucleostromal cells overexpress RANKL or TNF-related activated inducer cytokines, stimulate the recruitment of osteoclasts into osteoclast-like giant cells, and actively enable host bone absorption.^[2–4]^ Because bone destruction can lead to bone defects and pathological fractures, surgical treatment is often required, and there is no consensus on the ideal treatment. At present, the main surgical methods include extended curettage (EC) and segmental resection (SR). Some scholars have reported that the incidence of GCT in the proximal femur is about 5.5%,^[5]^ and it mainly affects the femoral neck and intertrochanteric, and rarely occurs in the subtrochanteric.^[6]^ We first report a unique case of proximal femoral GCT, a subtrochanteric lesion associated with femoral neck and intertrochanteric involvement. Although intramedullary nails are often used for bone destruction caused by metastatic tumors, they may cause metastasis to the distal femur. Based on the imaging grade, a defect size and patients’ wishes, the tumor managed by EC and reconstructed with a fibular strut allograft and long intramedullary interlocking nail was used for prophylactic fixation of fractures. The aim of the treatment was to restore hip function as much as possible and reduce the possibility of GCT recurrence and metastasis post-surgery.

## 2. Case report

### 2.1. Patient information

The patient, a 26-year-old man, came to our hospital for treatment of right hip pain more than 4 months ago, had no family history of similar diseases. Physical examination revealed swelling of the right hip joint, deep tenderness of the proximal femur, no obvious mass, increased pain during flexion and extension of the hip joint, and no significant restriction of movement.

### 2.2. Diagnostic assessment

**Figure 1.**
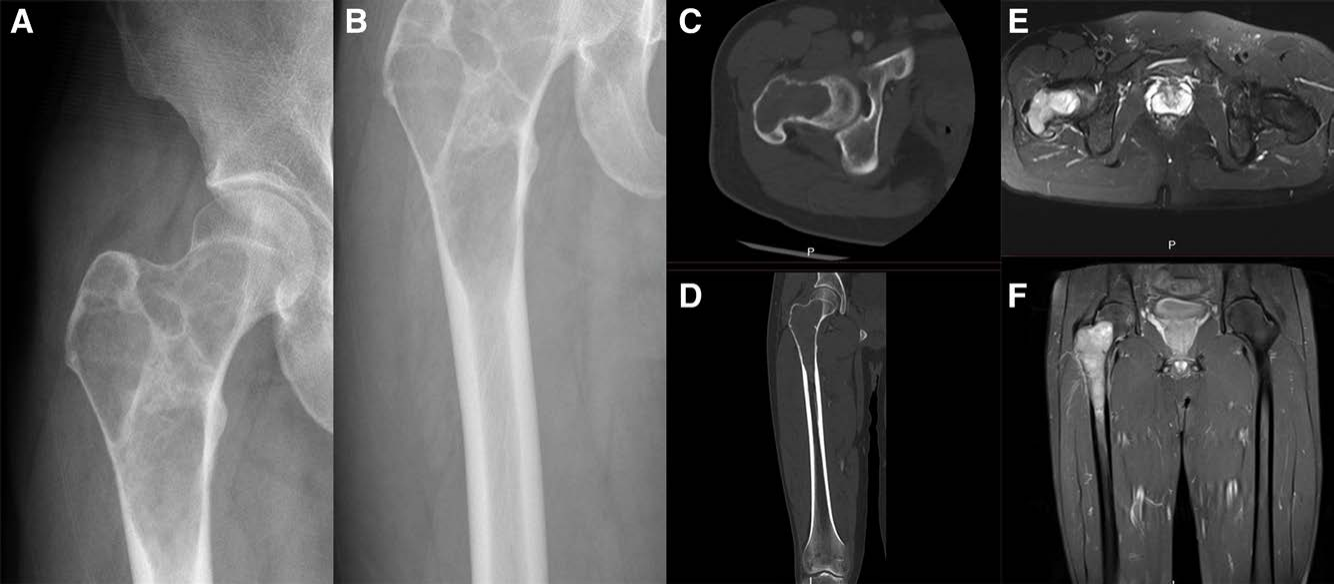
(A and B) Preoperative anteroposterior radiographs showed extensive cystic lesions in the subtrochanter, neck, and intertrochanter of the femur. (C and D) Preoperative computer-enhanced tomography showed no pathological fracture of the proximal femur, and the cortical bone was damaged but intact. (E and F) Preoperative magnetic resonance imaging showed that the lesion did not infiltrate the surrounding soft tissue.

Abdominal enhanced computed tomography (CT) demonstrated a large cystic lesion with a soapy appearance was found in the subtrochanter, neck, and intertrochanter of the femur, the cortical boundary of the proximal femur is intact and did not break through the bone cortex or penetrate into the surrounding soft tissue (Fig. 1). According to the Campanacci scale, the tumor was grade II. Our initial diagnosis was giant cell tumor of bone and it will eventually be confirmed by the postoperative pathological results. Based on the radiographic grade and the patient’s wishes, the patient underwent a completely new surgical approach for the treatment of primary tumors, and did not undergo disuzumab.

### 2.3. Therapeutic intervention

**Figure 2.**
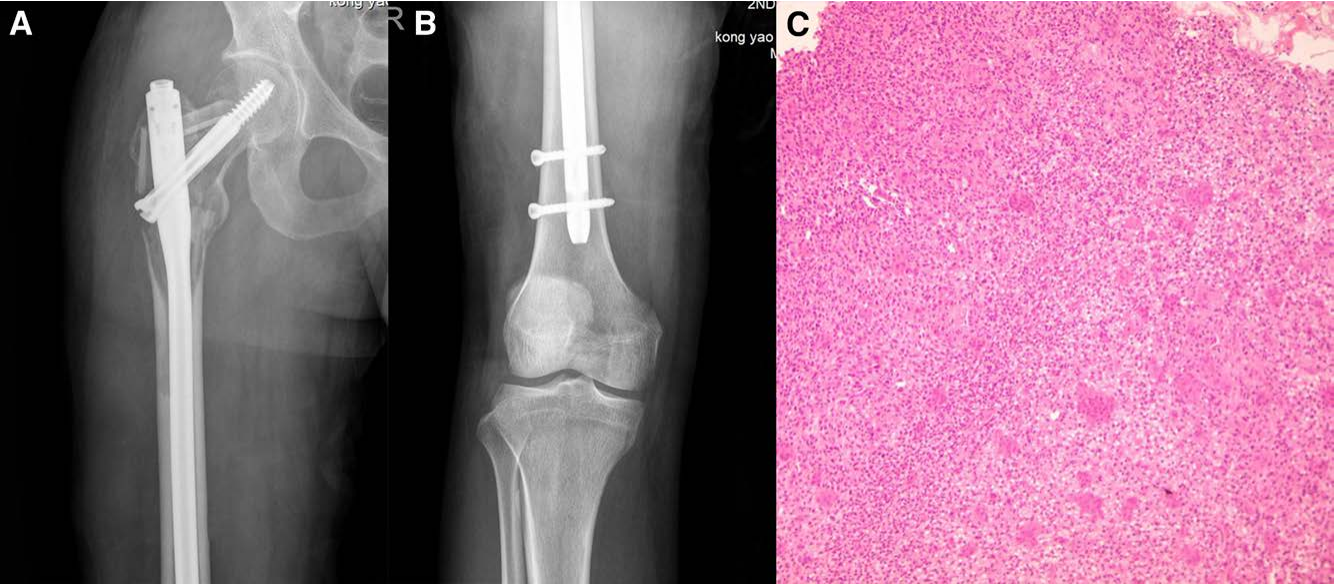
(A and B) Postoperative anteroposterior radiographs showed that the allograft fibula were in good position at 3 sites after intramedullary nail-fixation. (C) A tumor rich in giant cells (HE × 100).

The tumor of the femur managed by EC and reconstructed with a fibular strut allograft and long intramedullary interlocking nail was used for prophylactic fixation of fractures. During the operation, Watson–Jones approach was selected, anterolateral longitudinal incision of hip joint was performed, lateral femoris muscle and fascia lata were incised. The lateral femur muscle is gradually detached and retracted inwards by the electrotome, gradually exposing the lateral and anterior femur. The gluteus medius and gluteus minimus are severed backwards, and the gluteus medius and gluteus minimus are progressively separated to expose the greater trochanter of the femur and the femoral shaft. A circular incision of the joint capsule was made at the base of the femoral neck, and an elliptical bone window was opened above the femoral neck and greater trochanter. Dark red tumor tissue with abundant blood supply was found inside. Appropriate amount of tumor tissue was taken for intraoperative pathological examination. The tumor tissue on the wall of the cavity was removed by curettage, and the bone ridge was polished by high-frequency electric knife and high-speed drill. After repeated rinsing and cooling with normal saline, the remaining tumor cells on the wall of the cavity were inactivated by carbolic acid and anhydrous ethanol cotton ball, respectively. After the femoral bone marrow cavity was remeditated, 320 mm long femoral bone intramedullary nail was installed, 95 mm length diagonal nail was placed at the femoral head, and the distal end was fixed with 2 nails. Fresh and frozen fibula allograft grafts were artificially trimmed and firmly placed in femoral neck, lesser trochanter and greater trochanter respectively. The remaining cavity was grafted with autogenous iliac bone. Adduction, internal rotation, and flexion of the hip joint were examined without joint dislocation, and both lower limbs were kept equal length after surgery. The wound was thoroughly hemostatic and repeatedly rinsed with a large amount of normal saline. Gauze and surgical instruments were counted, a drainage tube was placed, deep fascia, subcutaneous tissue and skin were sutured in turn, and finally sterile dressing was pressurized. The intraoperative blood loss was 800 mL, the blood transfusion was 1000 mL, and the patient was returned to the ward after recovery from anesthesia. Postoperative radiographs showed that the allograft fibula were in good position at 3 sites after intramedullary nail-fixation, and the pathological diagnosis was giant cell tumor of bone (Fig. 2). Patients were discharged with instructions of no weight-bearing in 6 weeks, partial weight-bearing for 6 to 12 weeks, and full weight-bearing after 12 weeks.

### 2.4. Follow-up and outcomes

**Figure 3.**
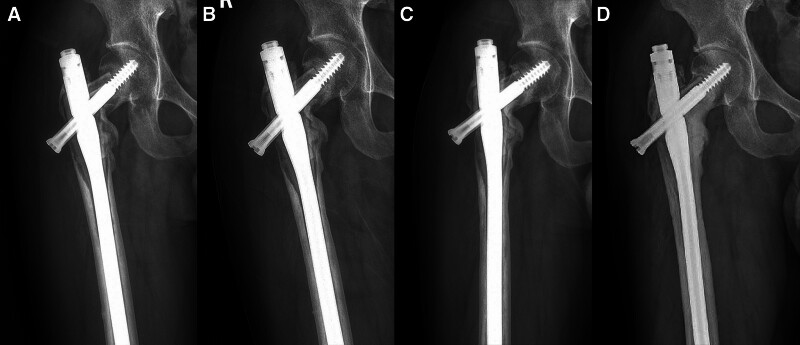
(A) Postoperative anteroposterior radiographs of the proximal femur showed bone resorption and atrophy at 3 months. (B) After 7 months of follow-up, the intramedullary nail showed signs of loosening. (C) After 12 months of follow-up, bone grafting began to heal and internal fixation gradually stabilized. (D) After 40 months of follow-up, the bone defect was healed and the intramedullary nail was stable in place.

The follow-up visits were completed at 1, 3, 6, 9, and 12 months within 1 year after operation, once every 6 months within 2 years, and then once a year. Bone graft absorption atrophy occurred around 3 months of follow-up, signs of loosening of intramedullary nails appeared around 7 months, bone graft healing began and internal fixation gradually stabilized around 12 months, and because the new coronavirus patient could not complete the follow-up, the bone defect had healed by about 40 months of final follow-up (Fig. 3). Chest radiographs showed no sign of lung metastasis throughout the follow-up period. The patient was 160 cm tall and always weighed about 55 kg. He had no limitation in his range of motion compared to his left hip, but he developed pain in his right hip after a long walk. The current MSTS score is 26.

## 3. Discussion

The etiology and pathogenesis of giant cell tumor of bone are not clear. Currently recognized large osteoclast-like giant cells are not tumor cells, while monocytes representing neoplastic components are believed to be derived from primitive mesenchymal stromal cells and have the characteristics of osteoblastic progenitor cells.^[7]^ However, multinucleated cells (which may exceed 50% of the total number of cells in the tumor) are derived from circulating monocytes, which are converted into osteoclasts in the bone environment. This conclusion is supported by studies on optical, ultrastructural and immunological markers.^[8]^ Khazaei et al^[9]^ illustrate through a series of studies that H3.3 G34W causes GCT by maintaining the transformation state of osteoblast-like progenitor cells, thus promoting tumor growth, pathological recruitment of giant osteoclasts and bone destruction. In 1940, Jaffe et al^[10]^ determined the histopathological grade of giant cell tumor of bone. Since stromal cells did not show malignant cytological characteristics, this grade had no predictive value for local aggressive behavior or metastasis.^[11]^ In 1987, Campanacci et al^[12]^ proposed the imaging grading system of giant cell tumor of bone, Campanacci grading, which played an important role in the selection of surgical methods for GCT clinical treatment. Open surgery is the most effective treatment for most GCT patients. Currently, surgical treatment options mainly include EC or SR, but the ideal treatment method is still controversial. Curettage can preserve joint function despite higher recurrence.^[13]^ Despite the low local recurrence rate of SR, a large number of research data show that the risk of postoperative complications is high,^[14]^ and the mechanical failure rate of young patients is higher than that of elderly patients,^[15,16]^ so the service life of artificial joint after surgery should also be considered. Without considering the influence of age factors, Yuhao Yuan et al^[17]^ conducted a retrospective study on 29 patients to compare the differences in local recurrence, reconstruction durability and postoperative function after treatment with EC or SR of proximal femur GCT. Complications in EC group (osteoarthritis, osteonecrosis) were significantly less than those in SR group (joint stiffness, infection, prosthesis loosening). Therefore, it is concluded that when the tumor is not extensively involved in the surrounding soft tissues, the articular surface is not damaged, and the pathological fracture is not significantly displaced, the EC should also be fully considered for the proximal femur GCT. Sakayama et al^[15]^ reviewed the records of 9 patients with proximal femur GCT, with an average age of 27.5 years.5 patients received curettage, and 2 patients had local recurrence after surgery. Hip arthroplasty was performed after recurrence, and the postoperative lower limb function evaluation was 93.3%. First choose the hip replacement surgery patients, 4 cases of all patients had no local recurrence, lower limb function evaluation was 93%. The results of this study showed that there was no significant difference in lower extremity function in patients with recurrent proximal femoral GCT who underwent hip arthroplasty for the second time compared with those who underwent hip arthroplasty for the first time. Zhang Xianghong et al^[18]^ conducted a retrospective study on 16 patients with proximal GCT prosthesis revision, with an average age of 46.6 years at the time of surgery. All of them received combination prosthesis replacement and allograft cortical support transplantation, with an average follow-up time of 46.3 months. No recurrence or metastasis was found, and the prosthesis was stable after surgery. Compared with the preoperative results, the average Harris hip joint score (70.6) was significantly improved (*P* < .05), and the lower limb length difference and musculoskeletal tumor social score were significantly improved (*P* < .05). The results suggest that modular prosthesis replacement and allograft cortical support transplantation may be selected for limb preservation in relatively young patients (mean age 46.6 years) with severe bone defects.

The invasiveness of GCT was mainly manifested in recurrence and lung metastasis. Multiple retrospective case series studies have shown that the local recurrence rate in patients treated with intrafocal curettage and local adjuvant therapy is 13% to 22%,^[19,20]^ which is significantly lower than that of curettage alone. Electroknife cautery and treatment of tumor cavity with phenol and anhydrous alcohol have been widely used in the inactivation of bone tumors. They kill residual tumor tissues through thermal damage and chemical burn, and can reduce the local recurrence of tumors.^[21–23]^ Bone defects caused by curettage of giant cell tumor of bone often require bone cement or bone graft to fill.^[24]^ Filling bone defects with bone cement can carry weight in the early stage, and the heating effect is conducive to killing residual tumor cells, which can reduce the local recurrence rate.^[19,25,26]^ Turcotte et al^[27]^ also reported a similar recurrence rate without the use of bone cement or other local adjuvant therapy in 2002. The recurrence rate mainly depends on whether the lesion can be completely curettled. In recent years, with the development of adjuvant therapy, the use of high-speed drill inactivation has become a more recognized adjuvant means.^[28,29]^ The main mechanism of its action is not only to inactivate the tumor hidden in the bone ridge by expanding the resection range while rotating at high speed, but also to further kill the tumor by generating high temperature. Jamshidi et al^[30]^ showed through their studies that the use of high-speed abrasive drilling significantly reduced the risk of local recurrence compared with traditional intrafocal curettage. Bone cement has been reported to increase the risk of joint degeneration.^[31–33]^ Bone grafts are associated with fracture risk and other potential complications such as avascular necrosis and osteoarthritis.^[6,34]^ When the bone defect is large, some scholars have proposed the use of fibula allograft, and studies have shown that it can provide better strength support, promote healing, and reduce the incidence of fracture.^[18,35]^ Studies have shown that lung metastasis is more common after local recurrence of giant cell tumor of bone.^[36,37]^ A multi-center retrospective study conducted by Jiang et al^[38]^ showed that the distal femur was the most prone to lung metastasis. Chest CT is usually recommended to evaluate for pulmonary metastasis. Lung metastases are generally benign, and a few can spontaneously resolve.^[39]^ Extended curettage and local adjuvant therapy can successfully treat potentially resectable local recurrent GCT, with less risk of increased complications,^[40,41]^ and generally a good prognosis.^[42]^ In June 2013, disuzumab was approved in the United States for use in patients with GCT who cannot be surgically removed or whose surgery is likely to cause serious complications, such as amputation or joint removal. Desumab, a fully human monoclonal antibody against RANKL, was initially shown to be beneficial in GCT patients in a groundbreaking Phase II trial.^[43]^ Histological results showed that desumab significantly reduced or eliminated giant cells, and also reduced the relative content of potentially neoplastic stromal cells, while promoting new bone formation.^[44]^ A Phase II trial of neoadjuvant therapy with desumab in 532 GCT patients,^[45]^ with a median follow-up time of 58.1 months in the total patient population, was conducted to evaluate the incidence of adverse events, including hypercalcemia (1%), atypical femoral fracture (1%), osteonecrosis of the mandibular bone (3%), hypophosphatemia (5%), and sarcomatosis (1%). The results of this study show that RANKL monoclonal antibody has a long-term control effect in GCT patients with unresectable and resectable tumors, and the overall risk benefit outweighs the harm.

The patient was only 24 years old, no obvious pathological fracture was found on CT, and Campanacci was classified as grade II. We conducted good communication with the patient before surgery, and the patient had a strong desire to preserve the joint. Therefore, we first determined the surgical method, and considering the large defect area, we recommended a fibular strut allograft and autogenous iliac bone transplantation. In the selection of internal fixation, we also considered the risk of metastasis of the distal medullary cavity of the intramedullary nail. The results of a study in 2021 showed that there is an extremely low likelihood of developing distal femoral metastases when isolated proximal femoral metastases are present.^[46]^ Arpornsuksant et al^[47]^ carried out a study to investigate the factors related to the progression of local metastases after intramedullary nail stabilization. The analysis suggests that the risk of experiencing local progression of tumor growth and reoperations after intramedullary nail stabilization seems to be low. Combined with the current literature, we finally decided to use intramedullary nails for fixation to prevent pathological fractures. We believe that with the clinical application of desumab and the increasing maturity of current methods for reconstruction of proximal femur,^[15,48,49]^ we should preserve the hip joint as much as possible for young patients and avoid hip arthroplasty, and hip rarthroplasty is indeed the best choice for patients with recurrent GCT. After 40 months of follow-up, although the bone defect finally reached bone healing, the hip function was good, and the tumor did not recur, there were signs of bone resorption atrophy and internal fixation loosening during the period, which did not affect the patient’s daily living activities.

## 4. Conclusion

Bone defects are often associated with EC of the proximal femur GCT. For young patients with imaging grade <3 who need limb salvage, fibular strut allograft and intramedullary nail-fixation are also an alternative treatment option for hip reconstruction after tumor surgery when the lesion involves the entire proximal femur. Due to the limited supporting strength of bone grafts and the long healing time of bone, it may be associated with a higher risk of loosening of internal fixation and fracture.

## Author contributions

**Investigation:** Kaipeng Jin.

**Supervision:** Quan Yao.

**Validation:** Quan Yao.

**Writing – original draft:** Jiashi Song.

**Writing – review & editing:** Bing Liu.
